# Comparative Study of the Expressions of Nuclear (∆EX3) and Cytoplasmic (2B) Survivins in Oral Squamous Cell Carcinoma and Oral Lichen Planus Using Real‐Time PCR

**DOI:** 10.1002/cre2.70123

**Published:** 2025-04-02

**Authors:** Maryam Amirchaghmaghi, Atessa Pakfetrat, Nooshin Mohtasham, Farnaz Mohajertehran, Mohammad Taghi shakeri, Elahe Vazavandi

**Affiliations:** ^1^ Oral and Maxillofacial Diseases Research Center Mashhad University of Medical Sciences Mashhad Iran; ^2^ Dental Research Center Mashhad University of Medical Sciences Mashhad Iran; ^3^ Social Determinants of Health Research Center Mashhad University of Medical Sciences Mashhad Iran; ^4^ Department of Oral and Maxillofacial Medicine, School of Dentistry Kerman university of Medical Science Kerman Iran

**Keywords:** gene expression, neoplasm, oral lichen planus, squamous cell carcinoma, survivin

## Abstract

**Objective:**

Survivin is used to determine the prognosis and clinical features of premalignant and malignant lesions. The aim of this study was to determine the correlation between the expression of survivin isoforms and clinical outcomes in oral lichen planus and oral squamous cell carcinoma.

**Materials and Methods:**

This cross‐sectional study examined 119 cases, including oral squamous cell carcinoma (SCC), oral lichen planus (OLP), and healthy margins of lesions. For all lesions, survivin expression was assessed quantitatively and qualitatively using real‐time polymerase chain reaction. The data were analyzed using SPSS 20.

**Results:**

The expression of survivin‐∆EX3 and survivin‐2B were quantitatively and qualitatively higher in SCC and OLP cases than in healthy mucosa (*p* < 0.05). The mean expression of survivin‐∆EX3 in erosive OLP (4.95 ± 4.41) was higher than that in nonerosive OLP (2.13 ± 3.32, *p* < 0.05). Moreover, the mean expression of both genes was significantly higher in different grades of SCC compared to healthy mucosa (*p* < 0.05). There was also a significant correlation between gene expressions (*p* < 0.001).

**Conclusion:**

The increased expression of survivin‐∆EX3 and survivin‐2B in OSCC correlates with tumor progression and advanced clinical stages, suggesting a potential prognostic role.

## 1 Introduction

Lichen planus refers to a group of chronic mucocutaneous lesions with different etiology and similar clinical appearance and histopathology (MichaelGlick et al. 2021). In the new WHO classification, oral lichen planus (OLP) is a premalignant lesion with the potential to transform into oral squamous cell carcinoma (SCC) (Tsushima et al. 2021).

In a study in 2008, the prevalence of lichen planus in patients referred to the Dental Clinic at the Faculty of Dentistry of Mashhad University of Medical Sciences in Iran was reported as 18.2% (Pakfetrat et al. 2009). Another study between 2002 and 2014 reported the prevalence of lichen planus in the Iranian population at 5.5% (Bakhtiari et al. 2017). In addition, it was more common in women than in men in the fourth and fifth decades of life (González‐Moles et al. 2021).

No clear etiology for lichen planus has been identified, although deficiencies of iron, zinc, calcium, folic acid, vitamins B6 and B12 have been observed in studies (Gholizadeh and Sheykhbahaei 2021; Chen et al. 2015). Folic acid as well as vitamins B6 and B12 play a role in reducing inflammation by converting homocysteine in to methionine, while their deficiency aggravates lichen planus (Chen et al. 2015). The main cause of OLP is the apoptosis of mucosal keratinocyte cells by cytotoxic T lymphocytes (MichaelGlick et al. 2021). Cytotoxic T cells induce apoptosis and activate the caspase cascade in keratinocyte cells through three mechanisms, namly binding to the keratinocyte TNFa receptor, binding via the Fas ligand receptor to the Fas receptor on the surface of keratinocytes, and perforation of the keratinocyte cell membrane by releasing enzymes (Granzyme B and Perforin) (Sugerman et al. 2002; Jan and Chaudhry 2019).

OLP is a premalignant lesion, which has the potential to transform into SCC. Therefore, early detection of premalignant and malignant lesions is of utmost importance for treatment (Tsushima et al. 2021).

**Figure 1:**
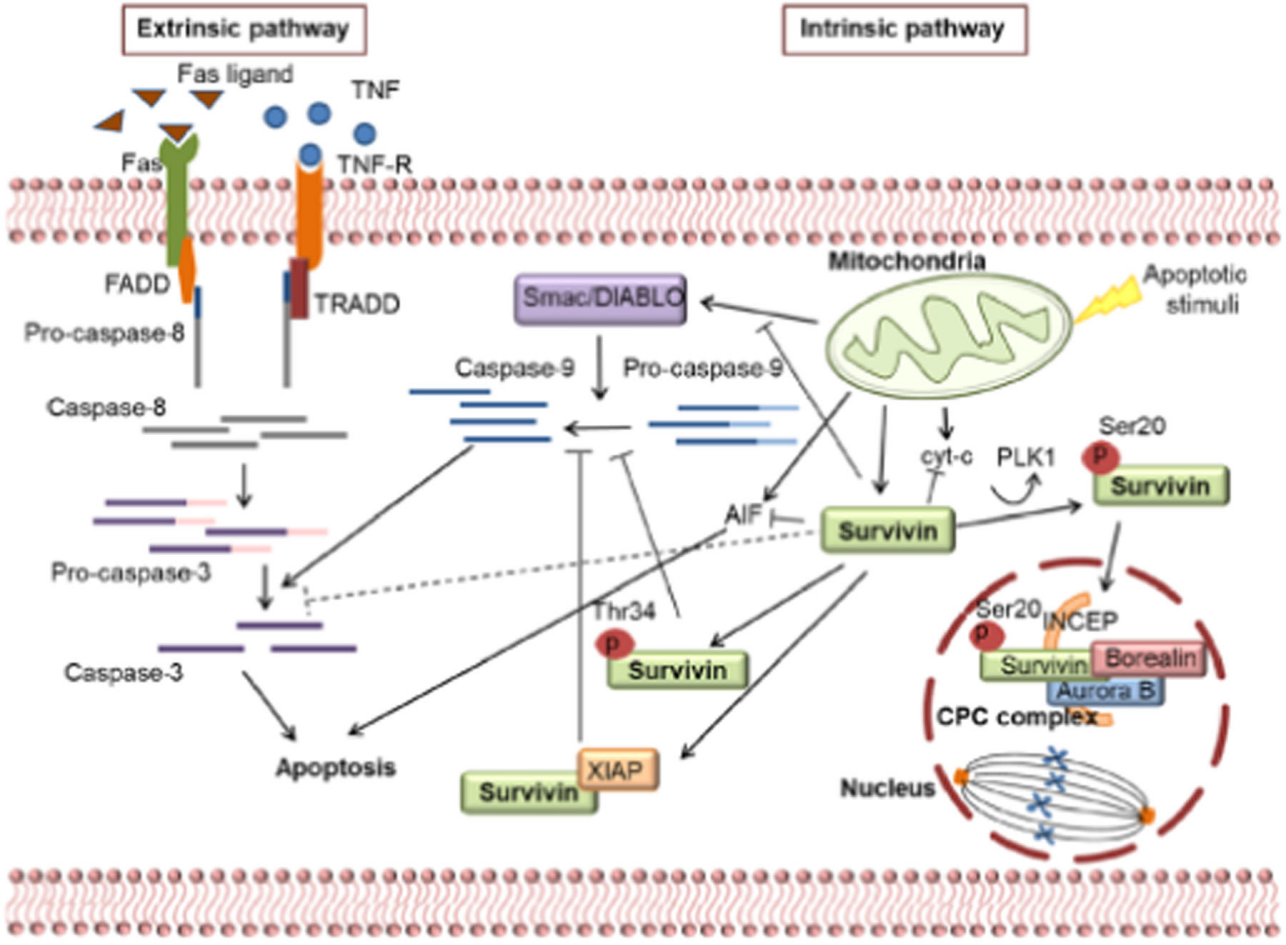
Roll of isotypes of survivin in nuclear and cytoplasm of a normal cell. Survivin has two different functions: 1. Apoptosis: some survivin variants cause activate apoptosis and others inhibit apoptosis. 2. Cell division and mitosis: survivin in the cytoplasm, after phosphorylation enters the nucleus, interacts with Aurora Borealin B kinase form CPC, and perform their role directly in nuclear division (Frassanito et al. 2019). The reason for including the areas adjacent to the tumors is the phenomenon of field cancerization and the fact that molecular changes are known even in the absence of dysplasia at the margins of the lesions.

Survivin acts in the cell nucleus, cytoplasm, and mitochondria (Raj et al. 2008) depending on the isotype involved in cell division, mitosis, and apoptosis (Figure 1) (Frassanito et al. 2019). Survivins identified to date include wild survivin (antiapoptotic), survivin‐2A (proapoptotic), survivin‐2B (proapoptotic), survivin‐∆EX3 (cell division and antiapoptotic), survivin‐3A (unknown function), and survivin‐3B (cell division and antiapoptotic) (Johnson and Howerth 2004; Knauer et al. 2007).

There are various studies on the role of survivin in cancer, such as stomach, kidney and ovarian cancers In the case of oral cancer, however, the results are conflicting. With regard to the transformation of premalignant lesions of the oral cavity into malignancy, several markers such as suvivin have been investigated. Nevertheless, few studies have been conducted on the type and role of survivin, although with conflicting results (Sakthivel et al. 2020; Lingam et al. 2021).

Compared to other methods, real‐time polymerase chain reaction (PCR) examines genes that express survivin in more detail, resulting in data that are more accurate. In the present study, the intensity of survivin expression in the nucleus and cytoplasm was examined using real‐time PCR along with the clinical features of SCC and OLP lesions. The aim was to determine and compare the expression of nuclear (∆EX3) and cytoplasmic (2B) survivin isoforms both quantitatively and qualitatively in OLP, OSCC and healthy mucosa.

## 2 Materials and Methods

This descriptive, analytical cross‐sectional study was conducted from December 2019 to November 2022 at the Department of Oral and Maxillofacial Diseases as well as the Pathology Laboratory and the Central Laboratory of the Faculty of Dentistry, Mashhad University of Medical Sciences, Iran. The study protocol was approved by the Ethics Committee of Mashhad University of Medical Sciences under the code IR.MUMS.DENTISTRY.REC.1400.028.

### 2.1 Methods

Formalin‐fixed paraffin‐embedded (FFPE) tissue samples were collected from 119 SCC and OLP cases. Samples were collected from the pathology archive on a simple random sampling basis, using a surgical blade other than a laser to biopsy the lesions. Healthy SCC margins and irritation fibromas (IF) were considered as control.

MichaelGlick et al. (2021) Inclusion criteria were patient demographic information such as age and gender, definitely diagnosed OLP and SCC lesions based on clinical and histopathological criteria, clinical information on OLP lesions (severity of lesions and their clinical appearance), as well as staging and grading of SCC lesions. Adjacent tumor areas were also included in the controls. The reason for this was the phenomenon of field cancerization and the fact that molecular changes are known even in the absence of dysplasia at the margins of the lesions.

Exclusion criteria were samples with lichenoid reaction, myoma samples with peripheral irritation and hyperplasia of the surface epithelium and amorphous infiltration in the connective tissue, OLP with dysplastic changes, samples with low‐quality extracted genes or with failed gene expression.

When studying gene expression patterns in cancer, selecting both tumor and nontumor tissue from the same individual is one of the best options as it eliminates most confounding factors. Therefore, tumor margin tissue from each patient was used as the control sample. This also helped to better understand how the tumor could affect surrounding tissue and identify subtle changes that indicate an increased risk of cancer. This approach highlights the complexity of tumor biology and the need for comprehensive assessment of both tumor and nontumor tissues in cancer research.

### 2.2 Assessments

#### 2.2.1 Thongprasom Score

We used Thongprasom scoring, a standard method for scoring OLP lesions to assess the clinical appearance of OLP lesions (Thongprasom 2018). Samples with erosive and bullous clinical appearance were included in the erosive group and those with atrophic, reticular, papular or plaque‐like appearance without erosion were included in the nonerosive group (Shang et al. 2020).

#### 2.2.2 Grading and Staging in OSCC Lesions

Definitely diagnosed SCC lesions were graded according to the degree of infiltration of cancer cells into the underlying connective tissue. The lesions were then divided into three groups, namely I, II and III based on histopathological criteria (Neville et al. 2016). Lesions were staged based on TNM classification and according to size, local lymph node involvement, and distant metastasis. To facilitate statistical analysis based on oncology sources, Stages 1 and 2 were classified as early and Stages 3 and 4 as advanced (Mohajertehran et al. 2019).

### 2.3 Real‐Time PCR

Isolation of total RNA was performed using High Pure RNA Paraffin Kit (Roche, Germany) according to the instructions. The purity and concentration of the extracted total RNA was determined using a NanoDrop 2000 spectrophotometer (NanoDrop Technologies, Wilmington, USA).

Complementary DNA (cDNA) was synthesized using the Thermo Scientific RevertAid First Strand cDNA Synthesis Kit (REF: 22701, Biotech, Addbio, Korea).

For quantitative expression of survivin, real‐time PCR (qPCR) was performed using SYBR Green Mix (TQ1110, SMOBIO, Taiwan) with the following targeting primers:

Survivin ∆EX3 Forward: 5′‐GACGACCCCATGCAAAG‐3′,

Survivin ∆EX3 Reverse: 5′‐GTGGCACCAGGGAATAAAC‐3′,

Survivin 2B Forward: 5′‐GATGACGACCCCATTGG‐3′,

Survivin 2B Reverse: 5′‐TTATGTTCCTCTCGTGATCC‐3′,

GAPDH Forward: 5′‐TGCACCACCACCTGCTTA‐3′, and

GAPDH Reverse: 5′‐GATGGCATGGACTGTGGTCAT‐3′.

The relative expression of RNA was calculated using the Ct values. The expressions for nuclear (∆EX3) and cytoplasmic (2B) survivins were normalized to the GAPDH reference gene. Gene expressions were compared based on the 2^−ΔCT^ method. Primers were designed using the Primer Blast database and Runner Gene software.

Given the test method used in this study, the cutoff value of the desired gene was considered 2, in accordance with similar studies (Dalman et al. 2012). The gene expression was classified into two groups, namely low and high expression.

Tissue samples were collected from the identified tumors, with tissue edges taken as controls from the unaffected tissue areas. Incisions were made in the tissue edges with a surgical blade and the samples were carefully placed in appropriate containers.

After transporting the samples to the laboratory, the samples were fixed in formalin solution and then embedded in paraffin. Thin sections were cut from paraffin‐embedded samples and placed on microscope slides. Tissue sections were routinely stained with conventional dyes, that is, hematoxylin‐eosin (H&E). Stained tissue sections were then examined under a microscope and a pathologist assessed the presence or absence of cancer cells. The results of the microscopic examination were recorded. The tissue samples and reports were saved and archived for possible future use.

### 2.4 Statistical Analysis

The data were analyzed using SPSS 20.0. Descriptive statistics were used to describe the data, including centrality, dispersion, and frequency distribution. For quantitative data analysis, the normality of the data was determined using the Kolmogorov–Smirnov one‐sample test. In case of normality, Student's *t*‐test and one‐way analysis of variance were used; otherwise, Mann–Whitney *U* and Kruskal–Wallis tests were used. Pearson's chi‐square test was used for qualitative data analysis, and Fisher's exact test was used in cases where more than 20% of the expected frequencies were below five. The significance level was set at 0.05.

## 3 Results

### 3.1 Clinical and Demographic Information

This study included 29 samples of OLP, 44 samples of OSCC, and 46 samples of healthy tissue (16 healthy inflammatory fibrosis, and 30 healthy margins of SCC). The four groups were homogeneous in terms of gender (*p* = 0.7), but they differed significantly in terms of age (*p* = 0.02). The buccal mucosa was the most common site in OLP lesions (72%) and the tongue was the most common site of SCC lesions (42.9%). Thirteen OLP samples (44.8%) were erosive and 16 (55.1%) were nonerosive. Twenty‐eight cases (63.6%) of SCC lesions were in early stage and 16 cases (36.4%) were in advanced stage (*p* < 0.001). In addition, there were 14 samples (31.8%) of Grade I and 15 samples (34.09%) of Grades II and III (Table 1).

**Table 1:** Demographic information of the subjects in the study (*n* = 119).

Group	Gender (*N*) M/F	Age mean ± SD
SCC	19:25	57.48 ± 14.5*
OLP	9:20	49.93 ± 14.36
Healthy margin of SCC	6:10	59.63 ± 15.20
Healthy margin of IF	10:20	49 ± 16.14
Total	119	

*Note*: The difference in mean age between the SCC group and other groups was significant.

Abbreviations: IF, irritation fibromas; OLP, oral lichen planus; SCC, squamous cell carcinoma; SD, standard deviation.

*p < 0.005.

### 3.2 Comparison of Gene Expressions Between Groups

The mean expression of survivin‐2B in SCC (4.98 ± 4.96) was significantly higher than those in healthy margin of SCC (0.97 ± 1.27, *p* = 0.001), healthy margin of IF (1.17 ± 1.31, *p* = 0.001) and OLP (1.43 ± 1.99, *p* = 0.004) (Table 2). The percentage expression of high survivin‐2B in SCC (26.0%) was significantly higher than those in healthy margin of SCC (3.0%, *p* = 0.006), healthy margin of IF (5.0%, *p* < 0.001) and OLP (6%, *p* = 0.001) (Table 2).

**Table 2:** Comparison of survivn‐2B and survivin‐∆EX3 expressions in the study groups (qualitative and quantitative).

Group	2B exp. mean ± SD	2B low exp. *n* (%)	2B high exp. *n* (%)	∆EX3 exp. mean ± SD	∆EX3 low exp *n* (%)	∆EX3 high exp. *n* (%)
SCC	4.98 ± 4.96	18 (40.9%)	26 (59.1%)	14.26 ± 16.23	15 (34.1%)	29 (69.9%)
OLP	1.43 ± 1.99	23 (79.3%)	6 (20.7%)	3.39 ± 4.04	16 (55.2%)	13 (44.8%)
Healthy margin of SCC	0.97 ± 1.27	13 (81.3%)	3 (18.8%)	1.25 ± 1.63	13 (81.3%)	3 (18.8%)
Healthy margin of IF	1.17 ± 1.31	25 (83.3%)	5 (16.7%)	1.33 ± 1.34	25 (83.3%)	5 (16.7%)
			***p* < 0.001**			

*Note:* Quantitative analysis: Based on the Kruskal–Wallis test, the difference in mean gene expression in four study groups was significant (*p* < 0.001). Bonferroni's post hoc test was used for pairwise comparisons (*p* < 0.001). Qualitative analysis: Based on the chi‐square test, the difference in the percentage of gene expression in four study groups was significant (*p* < 0.001).

Abbreviations: IF, irritation fibromas; OLP, oral lichen planus; SCC, squamous cell carcinoma; SD, standard deviation.

Furthermore, the mean expression of survivin‐∆EX3 in SCC (14.26 ± 16.23) was significantly higher than those in healthy margin of SCC (1.25 ± 1.63, *p* = 0.001), healthy margin of IF (1.33 ± 1.34, *p* = 0.001) and OLP (3.39 ± 4.04, *p* = 0.004) (Table 2). He percentage expression of high survivin‐∆EX3 in SCC (69.9%) was significantly higher than those in healthy margins of SCC (18.8%, *p* = 0.001) and healthy margin of IF (16.7%, *p* < 0.001). In addition, the percentage expression of high survivin‐∆EX3 in OLP (44.8%) was significantly higher than that in healthy margin of IF (16.7%, *p* = 0.02) (Table 2).

The results of general linear and logistic regression models indicated a significant difference between groups in the quantitative and qualitative expression of 2B and ∆EX3 survivins, even after adjusting for the effect of age.

### 3.3 Gene Expression in OLP Samples

The mean expression of survivin‐∆EX3 in erosive OLP (4.95 ± 4.41) was significantly higher than that in nonerosive OLP (2.13 ± 3.32, *p* = 0.009) (Table 3). According to the results of the Kruskal‐Wallis test, there was a statistically significant difference in the quantitative expression of surviving‐∆EX3 at different Thongprasom scores (*p* = 0.049).

**Table 3:** Comparison of quantitative and qualitative distribution of 2B and ∆EX3 gene expressions in the OLP group according to clinical appearance.

Group OLP	2B exp. mean ± SD	2B low exp. *n* (%)	2B high exp. *n* (%)	∆EX3 exp. Mean ± SD	∆EX3 low exp. *n* (%)	∆EX3 high exp. *n* (%)
Erosive	2.14 **±** 2.76	9 (69.2%)	4 (30.8%)	4.95 ± 4.41	5 (38.5%)	8 (61.5%)
Nonerosive	0.84 **±** 0.69	14 (87.5%)	2 (12.5%)	2.13 ± 3.32	11 (68.8%)	5 (31.3%)
*p*‐Value				*p* < 0.05		

*Note:* The Mann–Whitney test was used for quantitative analysis of gene expressions (*p* < 0.05). Fisher's exact test was used for qualitative analysis of 2B expression (*p* < 0.05) and chi‐square test was used for that of ∆EX3 gene expression (*p* < 0.05).

Abbreviations: OLP, oral lichen planus; SD, standard deviation.

Nevertheless, the qualitative expressions of survivin‐2B and surviving‐∆EX3 showed no significant association with the clinical appearance or severity of OLP lesions.

### 3.4 Gene Expression in SCC Samples

#### 3.4.1 Survivin Expression in Different Stages

The advanced stages of the SCC group showed a mean survivin‐2B expression of 10.09 (±3.84), compared to the early stages of SCC, which had a mean of 2.06 (±2.57), (*p* < 0.001). Moreover, Fisher's exact test showed that, 35.7% of early‐stage SCC samples had qualitatively high survivin‐2B expression, while 100% of advanced‐stage lesions exhibited high survivin‐2B expression (*p* < 0.001).

Similarly, the advanced stages of the SCC group showed a mean surviving‐∆EX3 expression of 32.76 (±11.08) compared to the early stages of SCC, which had a mean of 3.69 (±6.16) (*p* < 0.001). Furthermore, 46.4% of early‐stage SCC samples had qualitatively high surviving‐∆EX3 expression, whereas 100% of advanced‐stage SCC lesions exhibited high surviving‐∆EX3 expression (*p* < 0.001).

#### 3.4.2 Survivin Expression in Different Grades

The Grade‐I SCC group showed a mean survivin‐2B expression of 3.28 (±4.29), compared to Grade‐II which had a mean of 5/19 (±5/72), and Grade‐III SCC, which had a mean of 6.63 (±4.57) (*p* = 0.04).

High expression of survivin‐2B was observed in 35.7% of Grade‐I SCC lesions, compared to 53/3% of Grade‐II and 86.7% of Grade‐III lesions (*p* = 0/01) (Table 4).

**Table 4:** Comparison of the quantitative and qualitative distribution of survivin‐2B and survivin‐∆EX3 expression in the SCC group with respect to the stage and grade of lesions.

Group SCC	2B exp. mean ± SD	low exp. 2B *n* (%)	high exp. 2B *n* (%)	∆EX3 exp. mean ± SD	low exp. ∆EX3 *n* (%)	high exp. ∆EX3 *n* (%)
Early stage	2.06 ± 2.57	18 (64.3%)	10 (35.7%)	3.69 ± 6.16	15 (53.6%)	13 (46.4%)
Advanced stage	10.09 ± 3.84	0 (0.0%)	16 (100.0%)	32.76 ± 11.08	0 (0.0%)	16 (100.0%)
Grade I	3.28 ± 4.29	9 (64.3%)	5 (35.7%)	9.93 ± 13.29	7 (46.7%)	7 (44.8%)
Grade II	5.19 ± 5.72	7 (46.7%)	8 (53.3%)	16.89 ± 19.72	7 (46.7%)	8 (55.2%)
Grade III	6.63 ± 4.57	2 (13.3%)	13 (86.7%)	15.67 ± 15.82	1 (6.7%)	14 (93.3%)
*p value	< 0/05					

*Note:* Lesion stage analysis: The Mann–Whitney test was used for the quantitative analysis of gene expression due to the non‐normality of the gene expression distribution (*p* < 0.05). Fisher's exact test was used for the qualitative analysis of gene expression (*p* < 0.05). Lesion grade analysis: For the quantitative analysis of gene expression, the Kruskal–Wallis test was used (*p* < 0.05). For the qualitative analysis of Survivin‐2B expression, the Chi‐square test was used. For the survivin‐∆EX3 expression, Fisher's exact test was used (*p* < 0.05).

Abbreviations: SCC, squamous cell carcinoma; SD, standard deviation.

## 4 Discussion

In this study, the highest expression of survivin‐2B and survivin‐∆EX3 were observed, both quantitatively and qualitatively, in OSCC lesions. The two gene expressions were also higher in OLP lesions compared to healthy control tissues. There was no significant increase in both gene expressions in erosive OLP lesions at higher Thongprasom scores compared to nonerosive cases at lower Thongprasom scores. Only the increase in the expression of survivin‐∆EX3 in erosive OLP at higher Thongprasom scores was quantitatively significant. In the SCC group, a significant association was found between the SCC grade and the quantitative and qualitative expressions of survivin‐2B as well as the qualitative expression of survivin‐∆EX3. At higher stages, quantitative and qualitative expression of both genes was significantly higher in the SCC group. In addition, the two gene expressions were correlated according to results of the Spearman's correlation test.

Similar to our study, Lipping et al. (2010), De De Maria et al. (2009), and Mishra et al. (2015) reported a significant increase in the quantitative and qualitative expressions of 2B and ∆EX3 survivin in OSCC samples compared to healthy mucosa and similar to our study. The higher expression of Survivin‐∆EX3 compared to survivin‐2B in SCC samples may be linked to P53 mutation, which enhance Survivin‐∆EX3 expression and contribute to tumor progression (Mishra et al. 2015). Survivin‐∆EX3 facilitates cell transition from the G1 to G2 phase and thus supports cell division and mitosis in SCC cancer cells (Mahotka et al. 1999; Sah and Seniya 2015; Liu et al. 2017). On the other hand, survivin‐2B plays a role in apoptosis, because it binds to wild survivin (an antiapoptotic survivin) in the cytoplasm and inhibits its action (Mishra et al. 2015). The increased expression of survivin‐2B in cancer cells can be due to inhibition of mitosis in cancer cell, but due to the mutation of P53 this is not successful (Mahotka et al. 1999; Sah and Seniya 2015). Similar to our results, Rupa Mishara et al. reported a significant positive correlation between the expression of 2B and ∆EX3 survivins (Mishra et al. 2015). In cancer cells, WT, ∆EX3 and 2B survivins have respectively the highest expressions (Mishra et al. 2015). This is perhaps one of the reasons for the increased expression of survivin‐2B to mitigate the inhibitory effects of apoptosis and aid in the destruction of cancer cells. When survivin‐2B is mutated, its function also changes and apoptosis does not occur, which extends the lifespan of cancer cells (Temme et al. 2005; Li et al. 2005). Lipping et al. used real‐time PCR and IHC to examine the relationship between the qualitative expression of survivin and the stage and grade of SCC lesions. They observed a significant increase in survivin expression with increasing tumor stage and grade (Liping et al. 2010). Similarly, in our study, we found that as the SCC stage increased, both gene expressions increased significantly, both quantitatively and qualitatively. With increasing grade, the quantitative expression of 2B and the qualitative expressions of 2B and ∆EX3 also increased significantly. In advanced tumors the balance between mitosis and apoptosis is disturbed in favor of higher mitosis and lower apoptosis (Qi et al. 2010). On the other hand, De Maria et al. (2009) used both real‐time PCR and IHC and quantitatively examined the expressions of 2B and ∆EX3 survivins in 22 OSCC samples and their healthy margins. They found no significant correlation between the grade and stage of the tumor and the quantitative expression of both survivin genes (De Maria et al. 2009). Mishra et al. qualitatively analyzed 2B and ∆EX3 expressions (fold change) in 75 samples of OSCC, healthy mucosa of SCC and 12 samples of healthy mucosa using real‐time PCR. They observed no significant association between the increase in survivin expression and the grade and stage. In view of the conflicting results of the studies, it is therefore worthwhile to examine four other survivin isotypes, in addition to the 2B and ∆EX3 survivins, which also play a role in mitosis and apoptosis, to determine the clinical behavior of tumors (Mishra et al. 2015).

Sugayana et al. (2016) and Sakthivel et al. (2020) used IHC and compared survivin expressions in OLP (with dysplasia and without dysplasia) and healthy mucosa. They found that survivin expression was significantly higher in OLP compared to healthy mucosa. In our study, although the two survivin expressions in OLP were quantitatively and qualitatively higher than in healthy mucosa, only the difference between the qualitative expressions of survivin‐∆EX3 in OLP and healthy margin of IF was statistically significant. Such a contradiction may be due to different assessment methods for gene expression (IHC and PCR). In addition, all sampes in the present study had no dysplasia, which may affect the results. The relative increase in survivin expression in OLP can be justified by the fact that the inflammatory cytokines secreted by T lymphocytes in the presence of chronic inflammation can expose P53 to mutation. Because P53 regulates survivin in cells, it increases survivin expression in the mutant state (Chaiyarit et al. 2009).

Sugayana et al. (2016) and Sakthivel et al. (2020) examined survivin expression in SCC lesions and compared it to that in OLP by immunohistochemistry, IHC) (Liping et al. 2010). Survivin expressions in the SCC group was significantly higher than that in OLP. In our study, the quantitative expression of 2B and ∆EX3 survivins and the qualitative expression of survivin‐2B were significantly higher in the SCC group than in OLP. As mentioned above, survivin‐∆EX3 plays a role in cell division and mitosis. Since cell division and mitosis are more frequent in cancer cells than in non‐cancer cells, the expression of survivin‐2B may increase to inhibit or compensate for such indiscriminate cell division (Mahotka et al. 1999; Sah and Seniya 2015; Liu et al. 2017). It is therefore expected that the expression of survivin‐∆EX3 and survivin‐2B is higher in SCC as a malignant lesion than in OLP. Hence, the two genes can be useful to predict malignant changes in OLP.

Using IHC, Lingam et al. (2021) compared survivin expressions in plaque‐like, reticular, and erosive OLPs. They showed that survivin expression was significantly higher in erosive OLP than the other two. In our study, the expression of both 2B and ∆EX3 survivins was quantitatively and qualitatively higher in the majority of OLP lesions with erosive appearance than in those with nonerosive appearance. However, only the increase in the quantitative expression of survivin‐∆EX3 in erosive lesions was statistically significant (with a mean of 4.95) compared to nonerosive lesions (with a mean of 0.84). In our study, the expression of survivin‐2B was quantitatively and qualitatively higher in lesions with higher Thongprasom scores (four and five) than in lesions with scores one and two, although the difference was not significant. Nevertheless, we found a significant relationship between the quantitative expression of survivin‐∆EX3 and Thongprasom score. Considering that survivin, in particular ∆EX3, is more strongly expressed in erosive OLP and at higher Thongprasom scores, it can be concluded that survivin expression may be a better marker for assessing the severity of OLP lesions and malignant changes. The reason for the increased expression of survivin in erosive OLP may be that erosive OLP is more inflammatory than nonerosive OLP. On the other hand, inflammation and the presence of various cytokines at the site can cause changes in P53, increase survivin expression and make cells more prone to inappropriate mitosis (Chaiyarit et al. 2009).

The limitations of this study were the small number of samples and the lack of samples with varying degrees of dysplasia. These can be addressed in further studies.

## 5 Conclusion

The significantly different expression of survivin‐2B and survivin‐∆EX3 in different tumor stages and grades shows that the two genes may be useful to predict the behavior of OSCC. The higher expression of survivins in advanced SCC compared to the healthy margin of SCC demonstrates the importance of the two genes for cell differentiation, tumorigenesis and the biological behavior of SCC.

In general, the increase in the expression of two genes in erosive OLP proves that there is a greater potential for malignant transformation. In addition, survivin‐∆EX3 had greater diagnostic value in OLP, while the expression of survivin‐2B was more related to the clinical behavior of SCC. Further studies are suggested to support these hypotheses.

## Author Contributions

Study conceptualization: Atessa Pakfetrat and Maryam Amirchaghmaghi. Data handling: Elahe Vazavandi. Experimental design: Atessa Pakfetrat, Maryam Amirchaghmaghi, Farnaz Mohajertehran, and Nooshin Mohtasham. Analysis and interpretation of data: Mohammad Taghi Shakeri. Provision of study materials and equipment: Elahe Vazavandi and Farnaz Mohajertehran. Study validation: Mohammad Taghi Shakeri and Nooshin Mohtasham. Supervision: Maryam Amirchaghmaghi and Atessa Pakfetrat. Data presentation: Elahe Vazavandi and Mohammad Taghi Shakeri. Draft preparation: Maryam Amirchaghmaghi, Atessa Pakfetrat, and Elahe Vazavandi. Study consultation: Nooshin Mohtasham. Writing and reviewing: Maryam Amirchaghmaghi, Atessa Pakfetrat, and Elahe Vazavandi. Project administration: Maryam Amirchaghmaghi and Atessa Pakfetrat. All authors approved the final version of this article.

## Ethics Statement

The study protocol was approved by the Ethics Committee of Mashhad University of Medical Sciences under the code IR.MUMS.DENTISTRY.REC.1400.028.

## Conflicts of Interest

The authors declare no conflicts of interest.

## Data Availability

The data sets generated during and/or analyzed during the current study are available from the corresponding author on reasonable request.
